# C-X-C Motif Chemokine Ligand 5 (CXCL5) Exhibits a U-Shaped Risk Profile for Mortality in Patients with Suspected Coronary Chest Pain

**DOI:** 10.3390/ijms27062744

**Published:** 2026-03-18

**Authors:** Dennis Winston T. Nilsen, Patrycja Anna Naesgaard, Volker Poenitz, Trygve Brugger-Andersen, Heidi Grundt, Annika Elisabet Michelsen, Pål Aukrust, Harry Staines, Thor Ueland

**Affiliations:** 1 Department of Clinical Science, University of Bergen, 5021 Bergen, Norway; 2 Department of Cardiology, Stavanger University Hospital, 4068 Stavanger, Norway; patrycja.anna.nesgaard@sus.no (P.A.N.); hagerman@online.no (T.B.-A.); 3 Department of Respiratory Medicine, Stavanger University Hospital, 4068 Stavanger, Norway; magnea.heidi.jonsdottir.grundt@sus.no; 4 Institute of Clinical Medicine, Faculty of Medicine, University of Oslo, 0318 Oslo, Norway; a.e.michelsen@medisin.uio.no (A.E.M.); paukrust@ous-hf.no (P.A.); thor.ueland@medisin.uio.no (T.U.); 5 Research Institute of Internal Medicine, Oslo University Hospital, Rikshospitalet, 0372 Oslo, Norway; 6 Section of Clinical Immunology and Infectious Diseases, Oslo University Hospital, Rikshospitalet, 0372 Oslo, Norway; 7 Sigma Statistical Services, Balmullo KY16 0BD, UK; harry.j.staines@gmail.com; 8 Thrombosis Research Center (TREC), Division of Internal Medicine, University Hospital of North Norway, 9037 Tromsø, Norway

**Keywords:** prognostic biomarkers, C-X-C motif chemokine ligand 5 (CXCL5), acute coronary syndrome (ACS), all-cause mortality, composite cardiovascular event (all-cause mortality or myocardial infarction or stroke)

## Abstract

CXCL5 is a platelet-derived chemokine which promotes inflammatory responses in neutrophils and monocytes through CXCR2. Previous studies on CXCL5 in atherogenesis are to some degree conflicting, with scarce outcome data following acute coronary syndrome. This study aimed to assess the utility and risk profile of CXCL5 as a prognostic marker of all-cause mortality at 5-year follow-up in patients hospitalized for chest pain of suspected coronary origin. We measured CXCL5 levels in platelet-poor plasma in 826 consecutive patients included in the “Risk Markers in the Acute Coronary Syndrome” (RACS) study (ClinicalTrials.gov Identifier: NCT00521976). Stepwise Cox regression models, applying quintiles, were fitted for the biomarker with all-cause mortality within 5 years as the dependent variable. At 5-year follow-up, 250 (30.3%) of the population had died; 34.5% in Quintile (Qt)-1, 32.1% in Qt-2, 23.5% in Qt-3, 29.1% in Qt-4, and 32.1% in Qt-5. Using Qt-3 as a reference, both the univariate and multivariable analysis showed a U-shaped association between CXCL5 and all-cause mortality. Univariate analysis: Qt-1 vs. Qt-3: HR 1.59 (95% CI 1.06–2.39), *p* = 0.026, and Qt-5 vs. Qt-3: HR 1.44 (0.95–2.18), *p* = 0.082, respectively. Multivariable analysis: Qt-1 vs. Qt-3: HR 1.65 (1.09–2.48), *p* = 0.017, and Qt-5 vs. Qt-3: HR 1.52 (1.00–2.30), *p* = 0.049, respectively. The U-shaped relationship was statistically strengthened, employing a composite endpoint consisting of all-cause mortality, MI or stroke. Our findings suggest that both too-low and too-high levels of CXCL5 may be harmful in patients admitted to hospital with chest pain of suspected coronary origin.

## 1. Introduction

Chemokines are low-molecular-weight inflammatory cytokines that play a crucial role in innate and adaptive immune responses [1]. By attracting leukocytes into the atherosclerotic lesion and inducing activation of endothelial cells and different leukocyte subsets within the lesion, chemokines play an important role in plaque progression and destabilization [2]. Indeed, several studies have shown an association between circulating levels of certain chemokines (e.g., CXC motif chemokine ligand 8 [CXCL8], CXCL10, CXCL16 and CC motif chemokine ligand 2 [CCL2]) and cardiovascular risk [3]. These molecules could also represent novel targets for therapy in atherosclerotic disorders [4]. However, the chemokine family constitutes a wide range of molecules, and the role of these mediators in atherosclerosis and their clinical consequences are still not fully understood.

CXCL5/epithelial-derived neutrophil-activating peptide 78 (ENA-78), derived from a variety of cells including monocytes/macrophages, endothelial cells, epithelial cells and particularly platelets, is a potent chemoattractant for neutrophils, T cells and monocytes, and plays a major role in angiogenesis through interaction with the chemokine receptor CXCR2 [3,5,6,7,8,9,10]. We have previously reported enhanced release of CXCL5 from peripheral blood mononuclear cells (PBMCs) and platelets in patients with coronary artery disease (CAD) when exposed to oxidized low-density lipoprotein (oxLDL) [11]. Moreover, Chen et al. suggested that CXCL5 could be a marker of subclinical atherosclerosis in type 2 diabetes mellitus (T2DM) [12], supported by the findings in a report by Wang et al. [13]. On the other hand, a protective role of CXCL5 in atherosclerosis is also suggested, potentially involving the impairment of foam cell formation [14,15]. Thus, to this end, the role of CXCL5 in atherosclerosis and development of myocardial infarction (MI) is somewhat unclear, and data on CXCL5 in relation to outcomes following admission for chest pain of suspected coronary origin is, to the best of our knowledge, lacking.

To further elucidate the role of CXCL5 in atherosclerotic disorders, we have investigated the association between CXCL5 and clinical outcomes during 5 years of follow-up in a chest-pain population admitted to hospital with clinically suspected acute coronary syndrome (ACS).

## 2. Results

**Figure 1:**
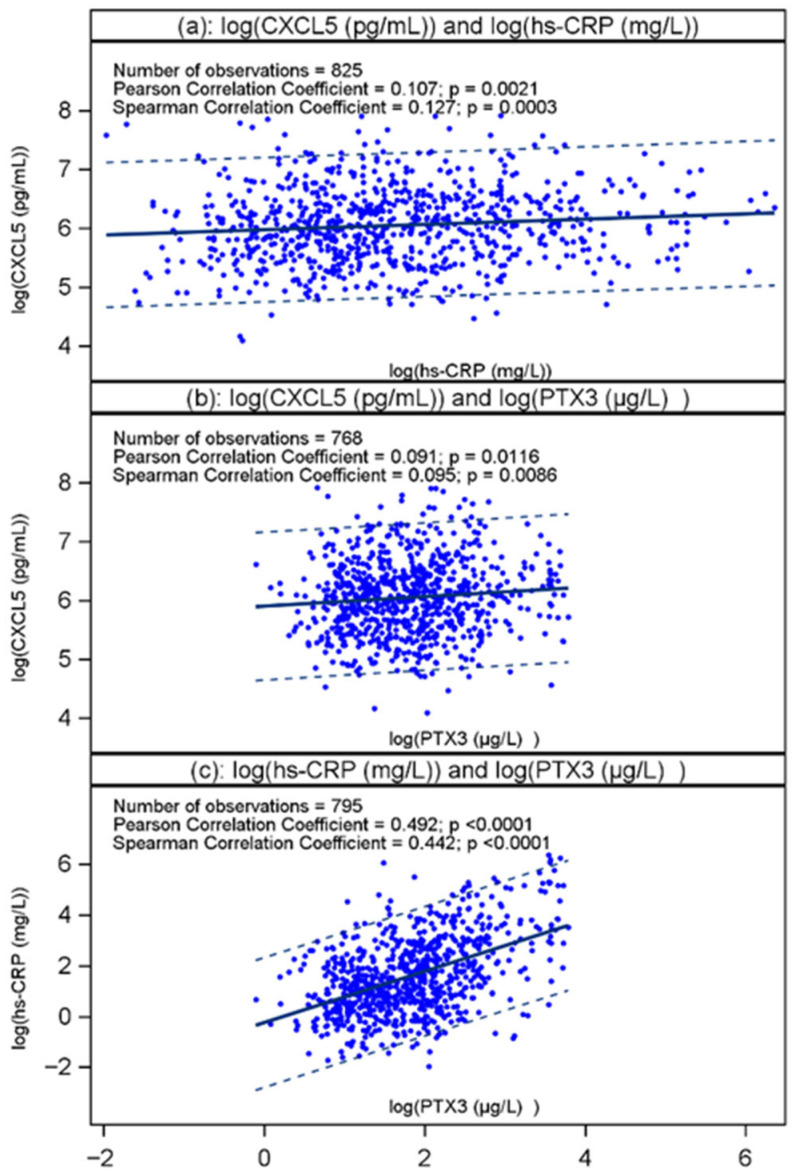
(**a**–**c**). Scatter plots based on the Pearson correlation coefficient between log_e_-transformed values of CXCL5 and the log_e_-transformed value of hsCRP (**a**) and PTX3 (**b**), respectively, and between the two latter (**c**), in 825, 768, and 795 RACS patients, respectively, admitted with chest pain of suspected coronary origin. The corresponding Spearman correlation coefficient is also shown.

We measured CXCL5 in plasma from 826 patients admitted with chest pain of suspected coronary origin, applying its log_e_-transformed value. A flow-chart is displayed in Supplemental Figure S1, and a histogram showing the distribution of the transformed values is depicted in Supplemental Figure S2. Table 1 shows the baseline characteristics of the study population according to quintiles of CXCL5. There was a gradual almost-5-times increase in CXCL5 from the lowest to the highest quintile, whereas eGFR, BNP and Pentraxin 3 (PTX3), the last being found to predict outcome in this cohort in males [16], were similar in all quintiles. There was, however, a significant increase in hsCRP and total cholesterol across the quintiles from Qt-1 to Qt-5. The proportion of patients with a negative TnT decreased significantly and a borderline increase in patients with detectable TnT was noted from the lowest to highest quintile. Comparing the extreme quintiles (Qt-1 vs. Qt-5), statin (38.8 vs. 24.9%) and beta-blocker (40% vs. 27.7%) use occurred more frequently in the lowest as compared to the highest quintile. As shown in Figure 1, CXCL5 correlated weakly with hsCRP and PTX3, respectively. Supplemental Figure S3 shows a scatter plot based on the Pearson correlation coefficient between log_e_-transformed CXCL5 and log_e_-transformed BNP. Neither the Pearson nor the Spearman correlation coefficient was significant at the 5% level, suggesting that CXCL5 does not reflect myocardial wall stress.

### 2.1. CXCL5 in Relation to All-Cause Mortality During 5 Years’ Follow-Up

**Figure 2:**
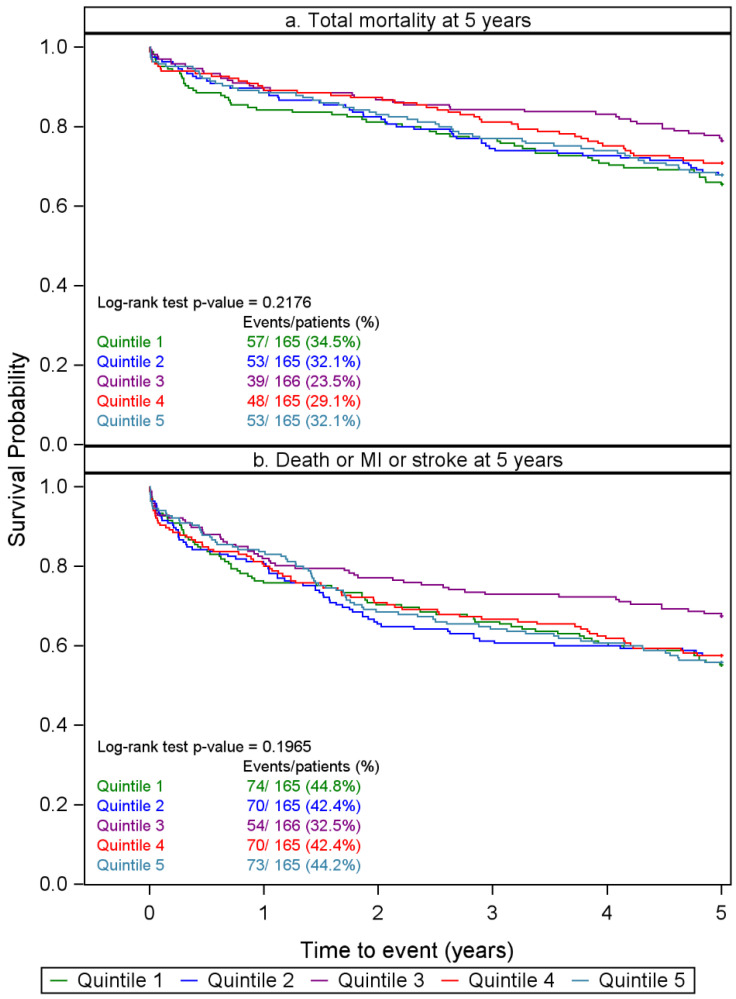
(**a**,**b**). Kaplan–Meier plots based on quintile median values of CXCL5 in relation to all-cause mortality and composite endpoint, respectively, at 5-year follow-up in patients admitted with chest pain of suspected coronary origin. (Composite endpoint = all-cause mortality or MI or stroke.) The multivariable model included potential covariables selected using a stepwise algorithm added to the model containing CXCL5 quintiles.

In this long-term follow-up study, all patients were assessed during a period of 5 years during which 250 patients had died. The risk of all-cause mortality was found to be U-shaped, employing cubic spline hazard ratios. The associations between CXCL5 quintiles and all-cause mortality during 5 years of follow-up are demonstrated in a Kaplan–Meier plot (Figure 2a). The log-rank test was non-significant (*p* = 0.218). In univariate analysis, however, the risk of all-cause mortality in Qt-1 was significantly higher (*p* = 0.026) than that of the Qt-3 reference and was also increased with borderline significance (*p* = 0.082) in Qt-5 as compared to Qt-3. Accordingly, we performed a quintile analysis of risk and chose the third quintile (Qt-3) as reference.

**Figure 3:**
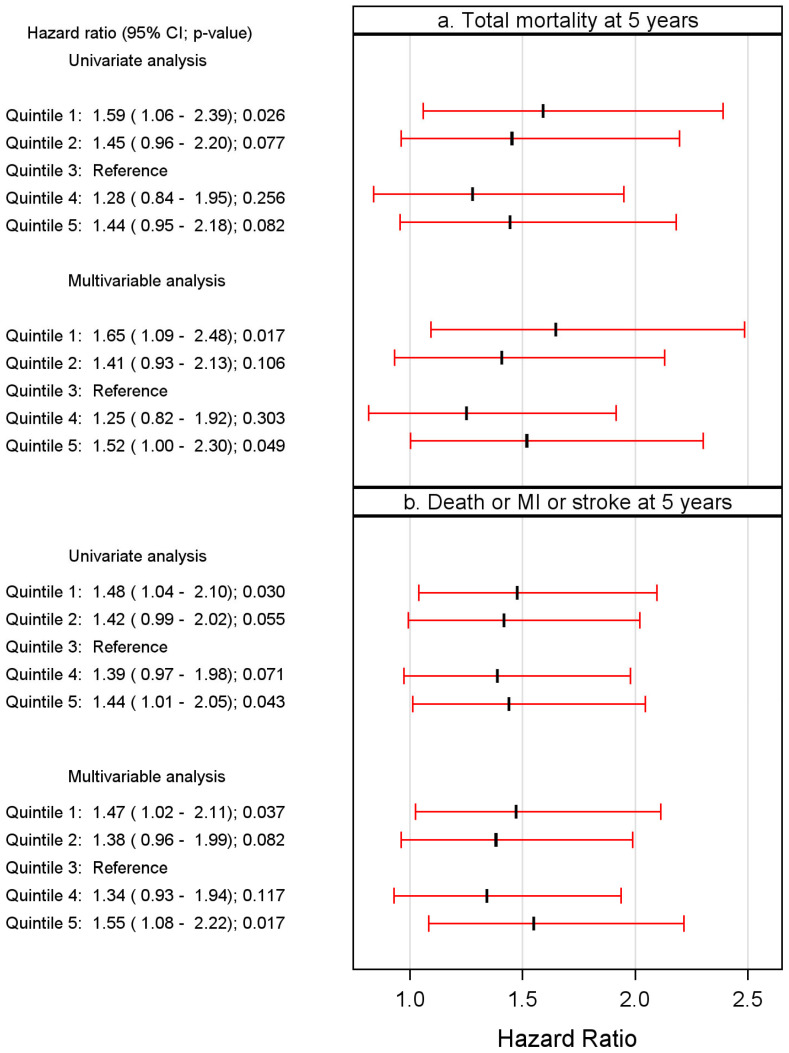
(**a**,**b**). Forest plots showing hazard ratios based on the univariate and multivariable analyses of the association between median quintile values of CXCL5 and risk of (**a**) all-cause mortality and (**b**) composite endpoint, respectively, at 5-year follow-up in patients admitted with chest pain of suspected coronary origin. (Composite endpoint = all-cause mortality or MI or stroke.)

**Figure 4:**
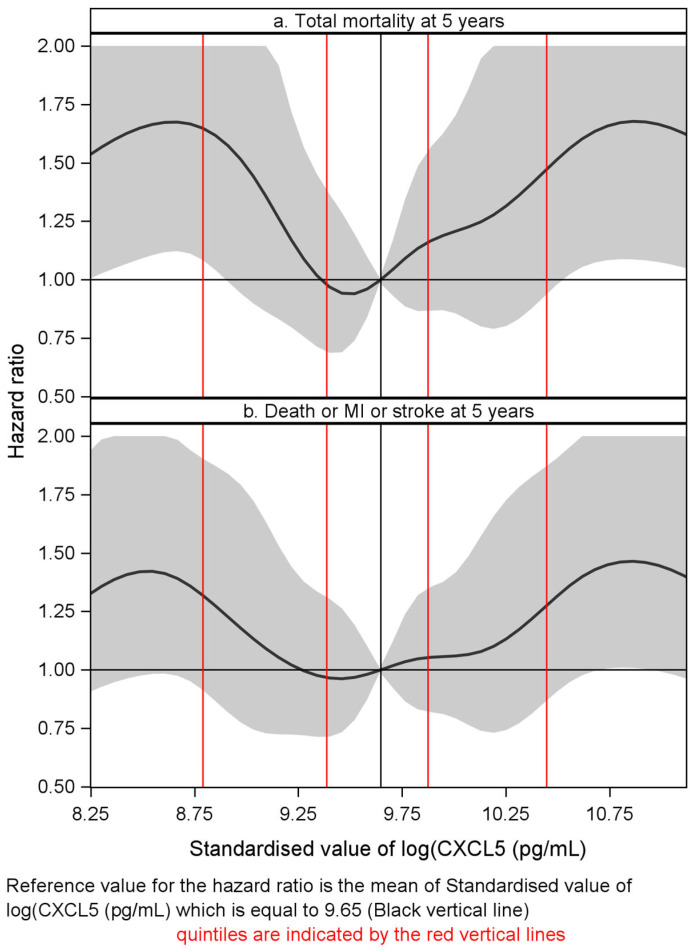
(**a**,**b**). Cubic spline interpolation of the quintile hazard ratios plotted against the mean of normalized baseline log(CXCL5) in relation to (**a**) all-cause mortality and (**b**) composite endpoint, respectively, adjusted for the covariates in the corresponding MVA model, at 5-year follow-up in patients admitted with chest pain of suspected coronary origin. Both plots demonstrate a U-shaped risk profile. (Composite endpoint = all-cause mortality or MI or stroke.)

There were four confounders in the multivariable analysis (age, BNP quartile, history of chronic heart failure and TnT > 10 ng/L). However, neither of the inflammatory markers (hsCRP nor PTX3) was selected in the stepwise algorithm. In the multivariable analysis, the U-shaped risk pattern was maintained, in which the CXCL5 levels in both the lowest (*p* = 0.017) and the highest quintile (*p* = 0.049) were significantly and independently associated with all-cause mortality as compared to the reference quintile (Qt-3). This is clearly demonstrated in a forest plot (Figure 3a). The risk of all-cause mortality remained U-shaped in a cubic spline plot adjusted for the covariates in the corresponding MVA model (Figure 4a).

### 2.2. CXCL5 in Relation to the Composite Endpoint During 5 Years’ Follow-Up

By 5 years, 341 patients had either died or suffered an MI or a stroke. The risk of the composite endpoint was also U-shaped, and the third quintile (Qt-3) was chosen as reference. Quintiles of CXCL5-associated risk for the composite endpoint are demonstrated in the Kaplan–Meier curves shown in Figure 2b. The log-rank test was non-significant, with a *p*-value of 0.197. In univariate analysis, the risk of the composite endpoint was significantly higher in Qt-1 (*p* = 0.030) and in Qt-5 (*p* = 0.043) as compared to the Qt-3 reference, whereas both Qt-2 (*p* = 0.055) and Qt-4 (*p* = 0.071) demonstrated a borderline significant increase.

In relation to long-time follow-up, six confounders emerged in the multivariable analysis (age, BNP quartile, history of chronic heart failure and TnT > 10 ng/L, plus smoking history and taking ACE inhibitors or ARBs). In the multivariable analysis, again, a U-shaped risk pattern was maintained, in which CXCL5 levels in both the lowest (*p* = 0.037) and the highest quintile (*p* = 0.017) were significantly and independently associated with the combined endpoint as compared to the third reference quintile. This is clearly demonstrated in a Forest plot (Figure 3b). The risk of all-cause mortality remained U-shaped in a cubic spline plot adjusted for the covariates in the corresponding MVA model (Figure 4b). We did not conduct a specific analysis related to MI or stroke, as their individual numbers were considered too small for conclusive results.

### 2.3. CXCL5 in Relation to TnT Groups

**Figure 5:**
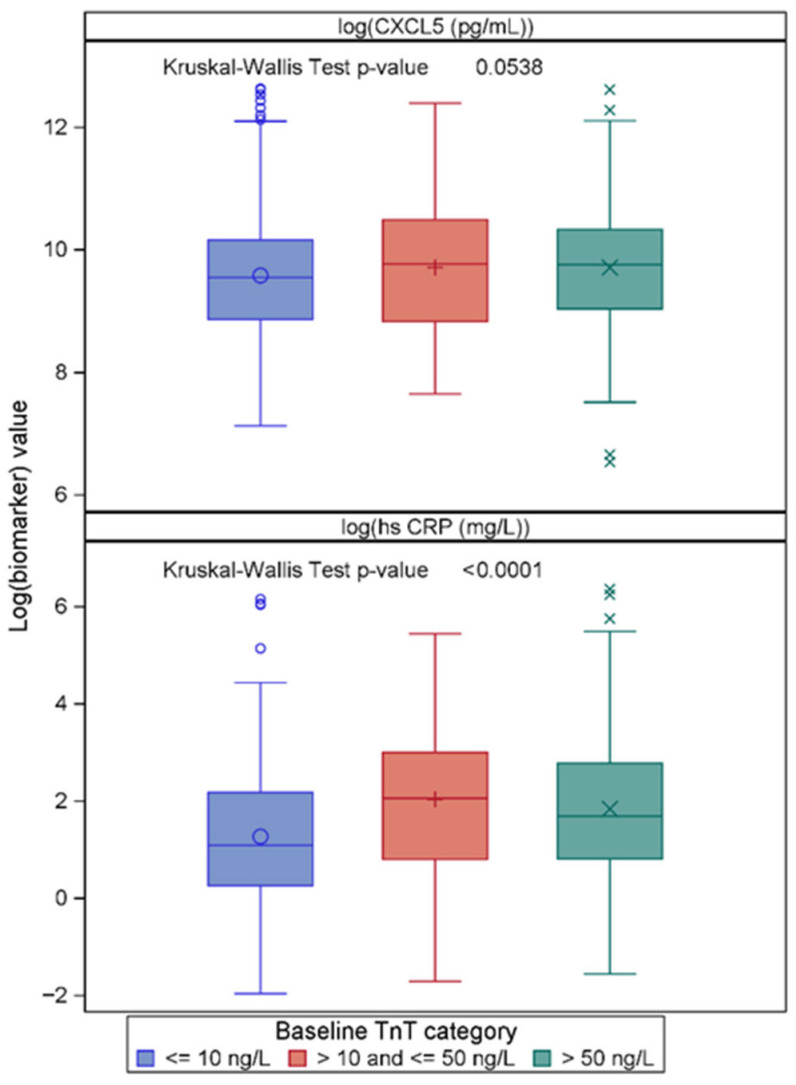
Box plot of log_e_-transformed CXC motif chemokine ligand 5 (CXCL5) and high-sensitivity C-reactive protein (hsCRP) concentrations, respectively, in three groups of patients categorized by their TnT values. The Kruskal–Wallace test was used to check for differences across the TnT categories.

The patient numbers and events in the TnT subgroups were considered statistically too low for the assessment of CXCL5-related risk. However, its levels across these subgroups were compared, together with those of hsCRP, to assess their discriminatory utility in relation to (1) suspected coronary chest pain with TnT < 10 ng/L [n = 381], (2) unstable angina with TnT values between 10 ng/L and up to 50 ng/L [n = 90], and (3) an acute MI with TnT levels > 50 ng/L [n = 355]. The results are shown in Figure 5. There was a statistically non-significant increase in CXCL5 (*p* = 0.054) along with increasing TnT levels, applying the Kruskal-Wallace test, whereas hsCRP increased significantly. Furthermore, a box plot by index diagnosis demonstrated no difference between patient groups, as shown in Supplemental Figure S4.

To further characterize our patients who displayed normal TnT values using the second-generation TnT assay and primarily classified as non-ACS, we measured high-sensitivity TnT in 29 randomly picked patients from this subgroup. Accepting an hsTnT level of <5 ng/L as negative, only nine patients in this subsample fulfilled the criteria of being negative, whereas those with elevated levels had values ranging from 5 to 35 ng/L, with a median of 14 ng/L. Thus, approximately 70% of patients originally defined as TnT-negative may have experienced an acute coronary event, and approximately 30% presented hsTnT values ≥ 14 ng/L, indicating the presence of an acute MI, defined according to hsTnT criteria [17].

## 3. Discussion

Data on the prognostic value of CXCL5 in relation to outcomes following admission for chest pain of suspected coronary origin are scarce. In the present study, the adjusted risk for all-cause mortality and the composite endpoint, respectively, showed a dual pattern of plasma CXCL5 levels in relation to outcome in the total population. Thus, the risk of all-cause mortality was increased in both the lowest and the highest quintile of CXCL5, using the third quintile as reference. Both extremes of the quintiles were independently associated with risk of death, suggesting a dual role of CXCL5 in atherosclerotic-related disease. This pattern may potentially reflect some previous studies describing both protective, i.e., modulation of macrophage phenotype and enhancement of cholesterol efflux [14], and harmful, i.e., enhancing neutrophil recruitment and plaque destabilization [18], effects of CXCL5, respectively. Moreover, whereas hsCRP and PTX3 are highly correlated, CXCL5 is only weakly correlated with hsCRP and PTX3, and can hardly be characterized as an acute-phase reactant.

Our findings of a positive relationship between the third and fifth quintiles of CXCL5 and all-cause mortality may support a previous report demonstrating a positive association between CXCL5 and the extent of CAD, potentially also reflecting a local accumulation of CXCL5 in coronary plaques [13]. Moreover, previous findings of an oxLDL-induced release of CXCL5 in PBMCs and platelets in patients with CAD could further support such a notion [11]. Furthermore, Zineh et al. identified a −156 G > C (rs352046) single-nucleotide polymorphism in the CXCL5 promoter that was associated with elevated plasma CXCL5 levels, and notably, this genotype was associated with increased mortality following ACS [19].

On the other hand, it has also previously been reported that increased CXCL5 expression in a mouse model did not increase neutrophil infiltration, whereas inhibition of CXCL5 expression induced a significant macrophage foam accumulation in murine atherosclerotic plaques [14]. Those findings would suggest a protective role of CXCL5 in atherosclerosis, as also claimed in a clinical report demonstrating a negative association between CXCL5 and CAD severity in 200 patients undergoing diagnostic coronary angiography [15].

The U-shaped association between CXCL5 and all-cause mortality requires further attention, and this is the first clinical outcome study that supports previous preclinical studies suggesting both protective and harmful effects of CXCL5 in relation to CAD and its clinical outcome. As the observed associations in the current report may reflect complex or unmeasured confounding, reverse causality, or unrelated biological processes, only assumptions can be made when comparing our data with those of previous reports.

The reasons for these dual effects are at present not clear. CXCL5 competes with other CXC chemokines in its class for their common receptor, CXCR2 [7,20]. Their affinities differ, not only in relation to the individual chemokine, but also in relation to whether the chemokines appear as monomers or dimers/oligomers, the latter being more strongly bound to the receptor [7]. Theoretically, considering dimers as blockers and monomers as activators of CXCR2, receptor accessibility may be increased at low concentrations of CXCL5 depending on the status of other chemokines and general immune response. At higher concentrations of CXCL5, more of its dimers are formed. This may result in less receptor availability, which could explain reports suggesting a protective role of CXCL5 [15]. However, at present, these issues are far from clear and need to be further investigated. Nonetheless, while previous studies on the dual role of CXCL5 are mainly based on preclinical models and in vitro experiments, the present study is, to the best of our knowledge, the first clinical study to support the double-edged role of CXCL5 in atherosclerosis, i.e., showing harmful associations of both low and high levels in the same patient cohort.

### Limitations

Patients with coronary suspected chest pain were selected clinically for this study, irrespective of troponin values. Inclusions and blood sampling were performed immediately after admission. All patients presented with anginal pain at admission, compatible with a coronary origin. For support of myocardial damage, we employed a modified “second-generation” TnT assay [16], known to be less sensitive than more up-to-date assays [20]. However, not only the AMI population, but also a large number of non-AMI patients presented with a history of established coronary heart disease. Furthermore, our use of hsTnT in a subsample of randomly picked patients from the group of patients classified as non-ACS demonstrated minor myocardial injury in approximately 70% of these patients. Nevertheless, the lack of a definite clinical diagnosis related to this group of patients should be regarded as a study limitation. Blood sampling for the measurement of the biomarkers was limited to one draw at hospital admission, and the association found will have to be related to the time of admission. Further studies would have to be done to investigate whether the association is affected by measurements in blood samples harvested during the course of hospital stays. Moreover, as BNP was found to be a confounder, heart failure may influence outcome and presumably also the CXCL5 levels, and the lack of left ventricular ejection fraction and other data on myocardial function is a limitation of the study. Furthermore, all subjects were recruited from a Norwegian population, and the management of these patients in 2002–2003 differs somewhat from the standard of care today, although all patients received both aspirin and a statin, and also revascularization by either percutaneous coronary intervention (PCI) or coronary artery bypass grafting (CABG) when clinically required according to national recommendations at the time of this study [16]. Moreover, demonstrating the ability of a biomarker to predict an outcome in clinical research may not apply to clinical practice due to the lack of automated instruments and assays with established reference ranges. Therefore, our results may not necessarily be generalizable and should be regarded as hypothesis-generating.

## 4. Materials and Methods

### 4.1. Study Design and Patient Population

Patients admitted to the emergency department with chest pain of suspected coronary origin were included consecutively in “Risk Markers in the Acute Coronary Syndrome (RACS)” (ClinicalTrials.gov Identifier: NCT00521976; approval date: 27 August 2007) [16]. The study was performed at Stavanger University Hospital, Norway, and included 871 patients recruited from November 2002 until September 2003.

The exclusion criteria were age < 18 years, unwillingness or incapacity to consent, and prior inclusion. An acute MI was defined by a Troponin-T (TnT) cut-off value of 50 ng/L, employing a rule-in-rule-out MI protocol based on blood samples harvested at baseline and 6–7 h after admission, and additional information from ECG readings was collected for further classification of the MI, as previously described [16]. Clinical follow-up data were based on hospital and public registries and by telephone interview at 30 days and 6, 12, 24 and up to 84 months, and any additional information was obtained from general practitioners and nursing homes [16]. Mortality beyond 2 years was essentially provided from death registries and limited to all-cause mortality [16]. Baseline data including demographics, smoking habits, clinical history, and general laboratory characteristics were collected at hospital admission.

### 4.2. Ethics

Written informed consent was required for participation. The study was approved by the Regional Board of Research Ethics and by the Norwegian Health Authorities, approval code: 118.02. The latest approval was by the Regional Board of Research Ethics, approval code 5764; approval date: 7 November 2024. It was conducted in accordance with the Helsinki Declaration of 1971, as revised in 1983.

### 4.3. Blood Sampling and Laboratory Measurements

Blood samples were collected by direct puncture of an antecubital vein immediately following hospital admission, applying a minimum of stasis, and an additional sample for measurement of TnT was drawn 6–7 h later. Centrifugation was performed for 15 min at 2000 *g* at 20 °C to obtain platelet-poor plasma, which was stored in aliquots as ethylene diamine tetra-acetic acid (EDTA) plasma and citrated plasma, respectively, at −80 °C. Routine tests, including for high-sensitivity C-reactive protein (hsCRP), TnT, brain natriuretic peptide (BNP) and estimated glomerular filtration rate (eGFR), were analyzed by the Medical Biochemistry Department at Stavanger University Hospital. TnT was measured with a modified second-generation assay. To obtain additional information with regard to myocardial injury in patients with TnT levels ≤ 10 ng/L, we randomly picked a subsample for measurement of high-sensitivity troponin-T (hsTnT), which was analyzed at the Medical Biochemistry Department at the National Hospital, Rikshospitalet, Oslo, by an electrochemiluminescence immunoassay (Elecsys 2010 analyzer, Roche Diagnostics, Basel, Switzerland).

### 4.4. Measurement of CXC Motif Chemokine Ligand 5 (CXCL5)

CXCL5 (pg/mL) was analyzed in duplicate in plasma from 826 patients, using an enzyme-linked immunosorbent assay (ELISA) with antibodies from R&D Systems (Catalog #: DY254, Minneapolis, MN, USA), and applying a 384-well format with a combination of a SELMA (Jena, Germany) pipetting robot and a BioTek (Winooski, VT, USA) dispenser/washer. An ELISA plate reader (BioTek) was used to measure absorption at 450 nm with wavelength correction to 540 nm. The intra- and inter-assay coefficients were <10%.

### 4.5. Statistics

Descriptive statistics are presented as medians with interquartile ranges (25th–75th percentiles) for continuous data and as numbers and percentages for categorical data. Differences in baseline characteristics were assessed by the Kruskal–Wallis test for continuous data and the Chi-squared test for categorical data. Due to a skewed distribution, Pearson’s and Spearman’s rank correlation coefficients were calculated between the log_e_-transformed values of biomarkers. For the cubic spline and Cox models, the log_e_-transformed CXCL5 value was normalized by dividing by the standard deviation (SD). Hence, the hazard ratios presented in the Results section are 1-SD on the log scale. The lack of prior knowledge on the nature of the CXCL5 effect suggested using a reasonable number of knots to add flexibility. The cubic split plot supported the use of quintiles and quintile-3 as the reference. Thus, restricted cubic splines were fitted at eight knots located at percentiles; 2.5, 97.5 and six equally spaced values in between. The hazard ratios, using the mean value of the normalized log (CXCL5) as the reference, were plotted to visualize the shape of the hazard associated with CXCL5. For the composite endpoint, the times to the first death, MI and stroke were used. No patients were lost to follow-up, and patients without an event were censored at five years. The Kaplan–Meier product limits were used for plotting the times to events, and the log-rank test was used to test for the equality of the survival curves. Cox regression models, using CXCL5 quintiles, with quintile-3 as the reference level, were fitted using the clinical endpoint as the dependent variable. The Cox model was based on quintiles alone and not on cubic splines. Results are shown separately for the univariate (UVA) and multivariable analysis (MVA), respectively. The multivariable model included potential covariables [shown by an asterisk in the baseline table (Table 1)] selected using a stepwise algorithm added to the model containing CXCL5 quintiles, applying a *p*-value threshold of 0.05 for entry and 0.10 for removal. Statistics were performed using the statistical package SPSS version 25 (IBM Corp. Armonk, NY, USA). All tests were 2-sided with a significance level of 5% without multiplicity adjustment. 

## 5. Conclusions

The present study is the first to support a dual role of CXCL5 in relation to clinical outcome in patients with chest pain of suspected coronary origin, with both low and high levels demonstrating a significant association with all-cause mortality or a composite endpoint consisting of all-cause mortality, MI or stroke at 5-year follow-up. These findings demonstrate a double-edged sword nature of CXCL5 where both high and low levels may be harmful. Its negative association with events at low levels is of particular interest, as this supports previous reports claiming beneficial anti-atherosclerotic effects in murine experiments and in patients undergoing diagnostic coronary angiography, in conflict with other studies claiming the opposite, which is more in accordance with a traditional point of view.

## Figures and Tables

**Table 1:** Baseline characteristics in 826 chest-pain patients with suspected coronary chest pain, arranged in quintiles (Qt) of CXCL5. The asterisk indicates potential covariables included in the multivariable analysis.

Characteristics	Total *n* = 826	Quintile 1 *n* = 165	Quintile 2 *n* = 165	Quintile 3 *n* = 166	Quintile 4 *n* = 165	Quintile 5 *n* = 165	*p*-Value
Biomarkers							
CXCL5 (pg/mL)	422 (270–629)	196 (151–218)	295 (270–330)	422 (389–452)	574 (527–629)	947 (797–1270)	<0.001
PTX3 (µg/L)	5.8 (3.5–9.3)	5.2 (3.2–8.0)	5.6 (3.4–9.3)	5.7 (3.4–9.1)	5.8 (3.5–9.3)	6.6 (3.8–11.1)	0.157
Risk markers at baseline:							
Age (years) *	72.6 (59.0–81.4)	72.9 (59.8–82.5)	72.8 (57.9–82.6)	73.8 (61.2–80.7)	71.3 (58.5–80.1)	71.6 (57.3–81.4)	0.721
Males *n* (%) *	504 (61.02)	110 (66.67)	106 (64.24)	105 (63.25)	100 (60.61)	83 (50.30)	0.024
BNP (ng/L) *	97.5 (34.0–314.0)	115.0 (42.0–395.0)	99.0 (31.0–266.0)	99.0 (39.0–286.0)	84.5 (35.0–336.0)	88.0 (32.0–261.0)	0.530
hsCRP (mg/L) *	4.0 (1.7–13.5)	3.2 (1.5–9.1)	3.2 (1.4–10.5)	5.0 (1.9–16.0)	4.4 (2.0–15.7)	5.0 (1.9–17.0)	0.003
eGFR (mL/min/1.73 m^2^) *	63.1 (48.4–75.4)	60.5 (45.4–73.0)	63.2 (48.6–75.1)	63.8 (48.4–76.2)	63.9 (49.5–77.1)	63.8 (50.3–73.4)	0.532
TC (mmol/L)	5.2 (4.2–6.0)	4.8 (4.1–5.6)	5.1 (4.2–6.0)	5.2 (4.3–5.9)	5.0 (4.2–6.1)	5.6 (4.6–6.2)	0.002
TnT ≤ 10 ng/L *n* (%)	381 (46.13)	83 (50.30)	85 (51.52)	84 (50.60)	62 (37.58)	67 (40.61)	0.023
TnT > 10 and ≤50 ng/L *n* (%)	90 (10.90)	20 (12.12)	14 (8.48)	16 (9.64)	17 (10.30)	23 (13.94)	0.533
TnT > 50 ng/L *n* (%)	355 (42.98)	62 (37.58)	66 (40.00)	66 (39.76)	86 (52.12)	75 (45.45)	0.054
Risk factors:							
Smoking							0.134
Ex-smoker	210 (25.4)	38 (23.0)	33 (20.0)	42 (25.3)	52 (31.5)	45 (27.3)	
Current smoker	306 (37.1)	61 (37.0)	74 (44.9)	65 (39.2)	56 (33.9)	50 (30.3)	
Never smoked	310 (37.5)	66 (40.0)	58 (35.2)	59 (35.5)	57 (34.6)	70 (42.4)	
Hypertension *n* (%) *	348 (42.1)	63 (38.2)	68 (41.2)	64 (38.6)	79 (47.9)	74 (44.9)	0.317
DM type I *n* (%) *	8 (1.0)	3 (1.82)	1 (0.6)	1 (0.6)	2 (1.2)	1 (0.6)	0.731
DM type II *n* (%) *	106 (12.8)	14 (8.45)	22 (13.33)	20 (12.05)	27 (16.4)	23 (13.9)	0.294
TC > 6.5 mmol/L *n* (%)	126 (15.23)	22 (13.3)	22 (13.3)	21 (12.7)	30 (18.2)	31 (18.8)	0.343
BMI (kg/m^2^)	25.3 (22.9–28.0)	24.9 (22.7–27.3)	25.2 (22.5–27.5)	25.4 (22.9–28.0)	25.5 (23.2–28.4)	25.7 (23.2–28.8)	0.248
History of heart disease;							
Angina pectoris *n* (%) *	366 (44.3)	64 (38.8)	87 (52.7)	72 (43.4)	72 (43.6)	71 (43.0)	0.137
Myocardial infarction *n* (%) *	276 (33.4)	54 (32.7)	57 (34.6)	59 (35.5)	54 (32.7)	52 (31.5)	0.942
Previous CABG *n* (%) *	85 (10.3)	18 (10.9)	21 (12.7)	23 (13.9)	13 (7.9)	10 (6.1)	0.105
Previous PCI *n* (%) *	85 (10.3)	20 (12.1)	19 (11.5)	18 (10.8)	16 (9.7)	12 (7.3)	0.625
Heart failure *n* (%) *	221 (26.8)	43 (26.1)	52 (31.5)	47 (28.3)	40 (24.2)	39 (23.6)	0.477
Treatment prior to admission:							
ACEI or ARB *n* (%) *	282 (34.1)	51 (30.9)	62 (37.6)	60 (36.1)	53 (32.1)	56 (33.9)	0.693
Beta-blocker *n* (%) *	298 (36.1)	66 (40.0)	65 (39.4)	55 (33.1)	67 (40.6)	45 (27.3)	0.049
Statins *n* (%)	286 (34.6)	64 (38.8)	60 (36.4)	63 (38.0)	58 (35.2)	41 (24.9)	0.054
ASA *n* (%) *	316 (38.3)	68 (41.2)	64 (38.8)	64 (38.6)	64 (38.8)	56 (33.9)	0.744

Data are presented as median (interquartile range) or numbers (%). Abbreviations: CXCL5, C-X-C motif chemokine ligand 5. PTX3, Pentraxin 3. hsCRP, high-sensitivity C-reactive protein. BNP, B-type natriuretic peptide. eGFR, estimated glomerular filtration rate. TnT, troponin-T. TC, total cholesterol. DM, diabetes mellitus. BMI, Body Mass Index. CABG, coronary artery bypass grafting. PCI, percutaneous coronary intervention. ACEI, angiotensin-converting-enzyme inhibitor. ARB, angiotensin receptor blocker. ASA, acetylsalicylic acid. * The asterisk indicates potential covariates adjusted for in the multivariable analysis. In each MVA, there are 28 missing values due to missing BNP.

## Data Availability

The original contributions presented in this study are included in the article/Supplementary Material. Further inquiries can be directed to the corresponding author.

## Supplementary Materials

The following supporting information can be downloaded at: https://www.mdpi.com/article/10.3390/ijms27062744/s1.
