# Supplementary material for: C-X-C Motif Chemokine Ligand 5 (CXCL5) Exhibits a U-Shaped Risk Profile for Mortality in Patients with Suspected Coronary Chest Pain
Source: Int J Mol Sci. 2026 Mar 18;27(6):2744. doi: 10.3390/ijms27062744 (PMC13026667; doi:10.3390/ijms27062744)
Supplement: Supplementary file 1 [file ijms-27-02744-s001.zip › ijms-4180679-supplementary.pdf]

Supplementary File:

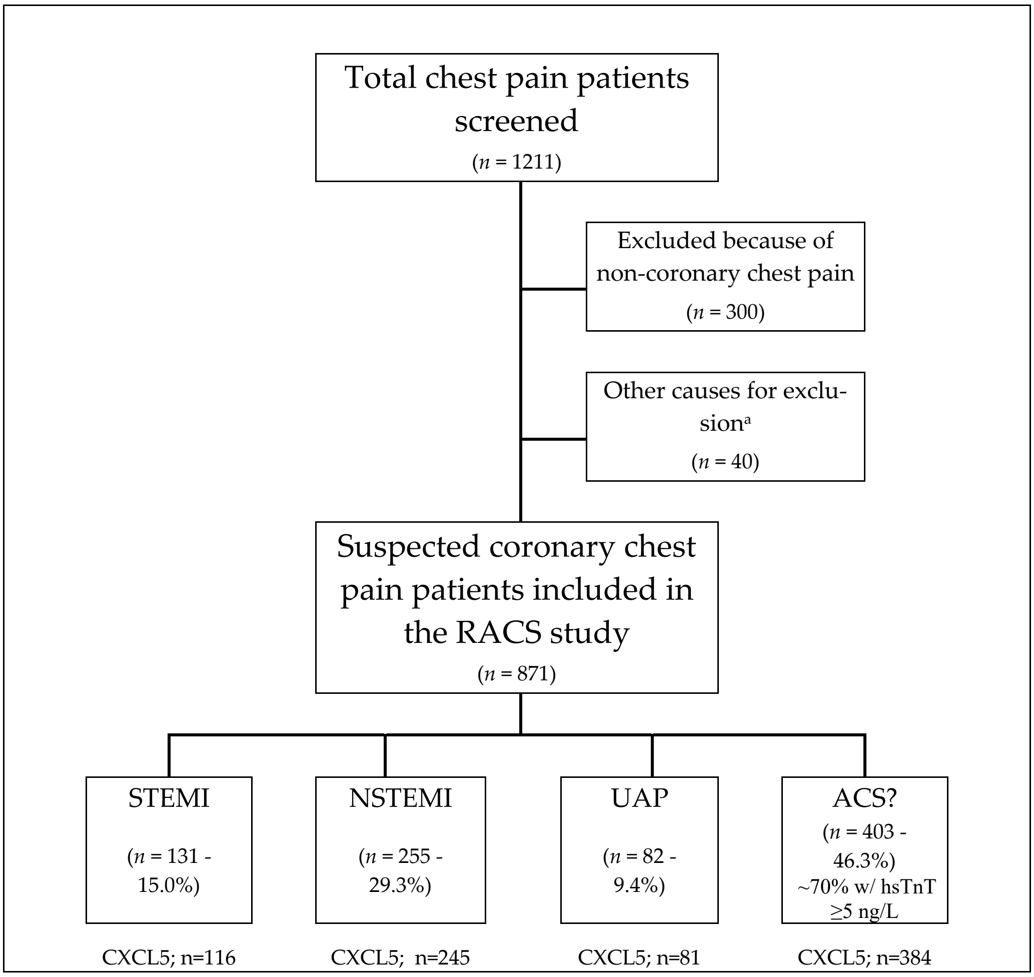

Figure S1. Flowchart RACS study and index diagnosis. [a]Exclusions: Sixteen patients were unable to cooperate due to various causes (confusion, coma, severe head trauma, severe cerebrovascular injury, severe dementia), five patients were unable to communicate due to language barriers, one patient was excluded because of age <18 years, four patients were excluded due to lacking initial test results, and fourteen patients did not wish to participate in the study. The flow chart also shows the number of patients with available measurements of CXCL5 in each diagnostic group. In the questioned ACS group, hsTnT was equal to 5 ng/L or elevated up to 35 ng/L. RACS = Risk Markers in the Acute Coronary Syndrome. STEMI = ST-segment elevation myocardial infarction. NSTEMI = Non-STEMI. UAP = Unstable Angina Pectoris. ACS = Acute Coronary Syndrome.

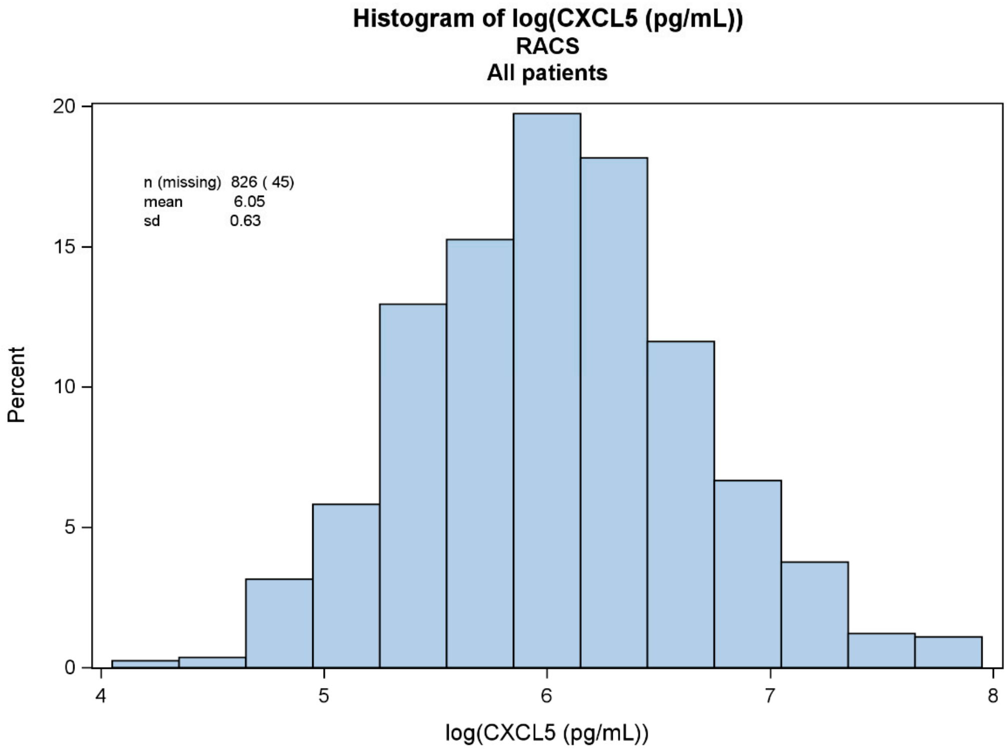

Figure S2. Histogram showing the distribution of log_e-transformed values of CXCL5 in 826 RACS patients admitted with chest pain of suspected coronary origin.

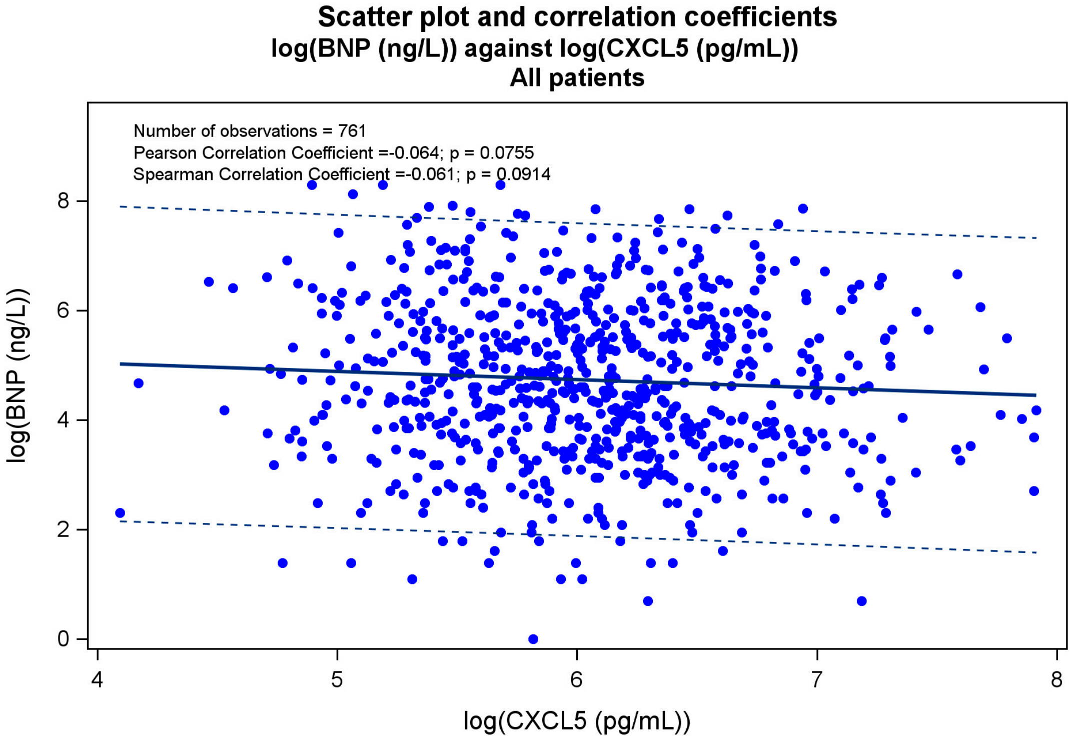

Figure S3. Scatter plot based on the Pearson correlation coefficient between $\log_e$-transformed CXCL5 and $\log_e$-transformed BNP.

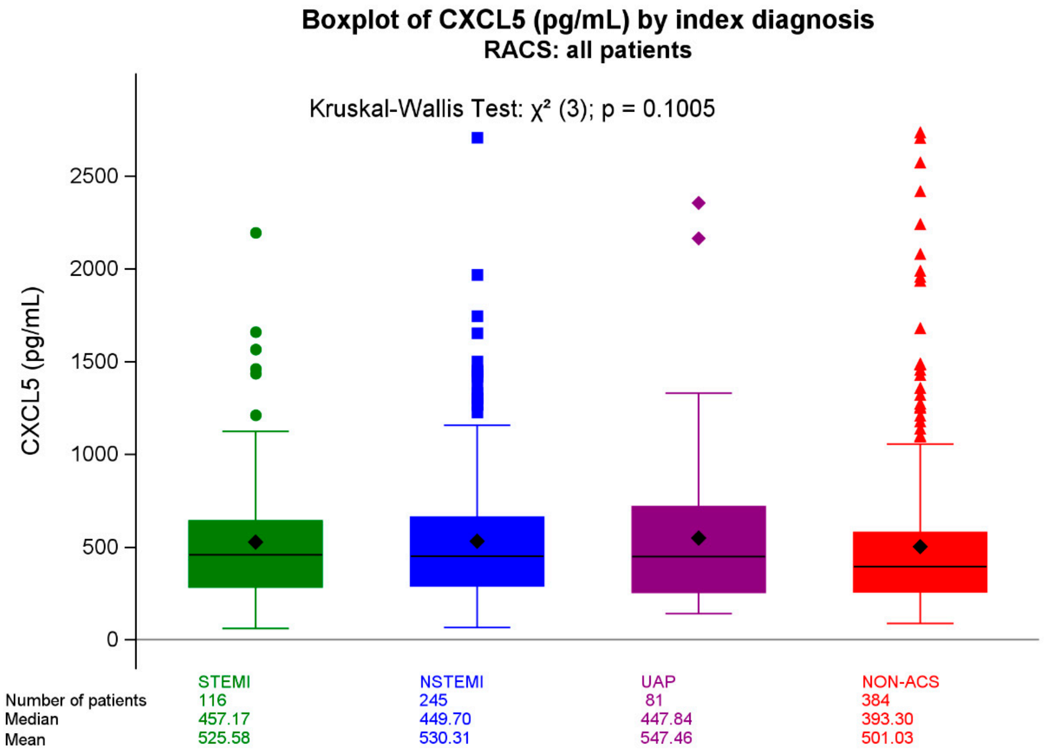

Figure S4. Box plot of CXCL5 by index diagnosis in the RACS study.
